# Applications of Nanofluids

**DOI:** 10.3390/nano11071716

**Published:** 2021-06-29

**Authors:** Mikhail A. Sheremet

**Affiliations:** Laboratory on Convective Heat and Mass Transfer, Tomsk State University, 634050 Tomsk, Russia; sheremet@math.tsu.ru; Tel.: +7-3822-529740

Nanofluids as a combination of base fluid and a low concentration of nano-sized particles of metal or metal oxides are used in different fields of human activity, including engineering devices in power and chemical engineering, medicine, electronics, and others. The main reason for such huge variety of nanofluid applications is the possibility, from one side, to enhance the heat and mass transfer due to the low concentration of nano-sized particles and, from the other side, to control the transport processes that can be used, e.g., in the drag delivery systems.

This Special Issue consists of twelve articles dedicated to nanofluid properties [1], the influence of nanoparticles on properties of other materials [2,3,4], as well as the possibility to enhance the heat transfer in different engineering devices using nanofluids, including the cooling of heat-generating elements in electronic devices [5,6,7,8], minichannel [9] and microchannel [10] optimization, and heat exchangers [11,12]. The objective of this Special Issue is to demonstrate the recent studies on nanoparticles and nanofluid applications in material treatment and engineering device optimization, which could be very useful for various specialists in mechanical and chemical engineering, physics, and mathematics. The following paragraphs include a brief overview of these mentioned papers with some innovative ideas.

Thus, we begin with a paper on an artificial neural network application for the prediction of the physical properties of nanofluids. Sadeghzadeh et al. [1] have synthesized the TiO_2_-Al_2_O_3_/H_2_O nanofluid and experimentally measured the specific heat capacity and thermal conductivity. After taking into account the opportunities of the neural networks, the multilayer perceptron structure has been employed to develop a model for the analysis of the thermal attributes of nanofluids. A good correlation can be achieved using the neural networks. Xie et al. [2] have analyzed features of the corrosive nature of a brass surface under the influence of nanofluids. It has been found that sodium dodecyl benzene sulfonate (SDBS) as a dispersant allows the formation of a protective film on the brass surface in simulated cooling water (SCW). At the same time, the addition of nanoparticles to the SCW-SDBS system can improve or degrade the protective properties of the film on the brass surface. Thus, an addition of negatively charged TiO_2_ nanoparticles does not allow the formation of an SDBS adsorption film, while in the case of positively charged Al_2_O_3_ nanoparticles, the properties of the protective film can be improved. García-Beltrán et al. [3] have studied opportunities of hybrid nanosystems for the improvement of the dynamic nonlinear optical effects. Thus, it has been revealed that the third-order nonlinear optical nature of metal/carbon nanosuspensions can be employed for developing dynamic nanoantennas. Zhu et al. [4] have analyzed novel opportunities to employ nanowires and nanotubes in chemical and bio-sensing engineering devices. The authors have analyzed the synthesis techniques for nanowires and nanotubes, sensing mechanisms of nanofluidic devices based on nanowires and nanotubes, and some applications.

Nanofluids are widely employed in different cooling systems for effective heat removal from electronics. Thus, Asadi et al. [5] have scrutinized the numerically convective heat transfer of copper oxide/water nanosuspension in a porous I-shaped chamber with a centered isothermal triangular block. Analysis has been performed using the Buongiorno nanofluid model with empirical correlations for effective viscosity and thermal conductivity. It has been ascertained that the orientation of an internal hot triangular block has an essential influence on the thermal pattern and flow structures within the chamber. Moreover, an addition of nanoparticles can enhance the heat removal from the local hot element. Bondareva et al. [6] have numerically investigated an opportunity to enhance the heat removal from the local heat-generating element under the influence of a heat sink filled with a phase change material. Five different phase change materials, enhanced with alumina nanoparticles, have been studied. It has been shown that n-octadecane, RT-80, and lauric acid are more effective materials for cooling enhancement. At the same time, an addition of alumina nanoparticles can slightly reduce the heat source temperature. An interesting numerical problem on the natural convection of alumina/water nanosuspension in a square chamber with inner solid block has been solved by Pop et al. [7] using the finite difference schemes and non-primitive variables. Such an approach allows a reduction in the computational time, but one of the main difficulties for such a formulation is a definition of the stream function value at the inner block. This problem was solved in the present study by employing the single-valued pressure along this block. As a result, a value of the stream function has been defined. Using the experimentally based correlations for nanofluid viscosity and thermal conductivity, the obtained outcomes have demonstrated that the growth of nanoparticle concentration reduces the energy transference strength. Using Fluent software, Jilte et al. [8] have numerically studied the heat transfer performance of a concentric channel thermal sink with an additional slot for the liquid. Taking into account the single-phase nanofluid approach with theoretical correlations for effective viscosity and thermal conductivity, it has been revealed that a rise in the flow rate characterizes a diminution of the maximum local temperature, while an inclusion of nanoparticles can also enhance the cooling effect.

An optimal design of minichannels can improve the heat transfer performance for various engineering devices. Thus, Ahmadi et al. [9] have studied a technique for the multi-objective optimization of minichannels in the case of alumina/water nanosuspension. The surface response methodology has been employed for the present analysis. Using the single-phase nanofluid approach with temperature-dependent properties, several numerical experiments have been performed. After, using statistical approaches, an analysis of the results has been conducted, and optimized parameters have been defined. Computational analysis of copper-water nanofluid flow and heat transfer in single and multi-channel heat sink has been performed by Khan et al. [10], employing Fluent software for the single-phase nanofluid model. The analysis has shown that the multi-channel system can increase the heat transfer rate, while an addition of nanoparticles also illustrates the energy transport enhancement. Alsabery et al. [11] have computationally examined the influence of a rotated inner cylinder on convective energy transport in a wavy differentially heated chamber filled with alumina-water nanofluid. Using the Buongiorno nanofluid model with effects of Brownian diffusion and thermophoresis, the authors have shown a possible energy transference enhancement with nanoparticle concentration. At the same time, the rotation of the cylinder and the waviness of the right vertical wall can intensify the convective heat transference. Ghalambaz et al. [12] have shown that using solid nanoadditives and twisted tape inserts allows the enhancement of the thermal performance of the double-pipe heat exchanger. Using Fluent software with two-phase nanofluid model, the authors have demonstrated the average Nusselt number increment for twisted tape inserts and alumina nanoparticles. 

These published papers have shown a huge diversity of nanofluid and nanoparticle applications based on detailed analyses using numerical and experimental techniques. 

## Data Availability

All data can be found in this paper or in papers cited here.
